# Accuracy and precision of dried urine spot method for the detection of *Schistosoma mansoni* circulating cathodic antigens in resource-limited settings

**DOI:** 10.1186/s40249-024-01183-7

**Published:** 2024-02-18

**Authors:** Abdallah Zacharia, Clemence Kinabo, Twilumba Makene, Huda Omary, George Ogweno, Faraja Lyamuya, Billy Ngasala

**Affiliations:** 1 Muhimbili University of Health and Allied Sciences, Dar es Salaam, Tanzania; 2 National Institute for Medical Research, Mwanza, Tanzania; 3 Neglected Tropical Diseases Control Program, Ministry of Health, Dodoma, Tanzania

**Keywords:** Accuracy, Point-of-care circulating cathodic antigen, Dried urine spot, *Schistosoma mansoni*, Sensitivity, Specificity

## Abstract

**Background:**

The World Health Organization recommends the use of Schisto point-of-care circulating cathodic antigens (Schisto POC-CCA) for screening of *Schistosoma mansoni* as it offers better sensitivity than microscopy. However, there are limitation facing the use of this method including timely availability of the test cassettes. The aim of this study was to determine the reliability of dried urine spot (DUS) method for collection of urine and detection of *S. mansoni* using Schisto POC-CCA cassettes in a resource-limited settings.

**Methods:**

A cross-sectional study was conducted between October and November 2022 among 250 primary school children in Sengerema District, northwestern Tanzania. *S. mansoni* CCA was detected in filter paper-based DUS, liquid urine using DUS Schisto POC-CCA (index), and direct urine Schisto POC-CCA (comparator) methods respectively. *S. mansoni* eggs in stool were detected using duplicate Kato-Katz (KK) method. The measures of accuracy were computed and compared between the index and comparator methods. The strength of agreement between inter-raters precisions was tested using Cohen’s kappa (*k*).

**Results:**

This study revealed *S. mansoni* prevalence rates of 28.8%, 54.0% and 50.8% by duplicate KK, direct urine Schisto POC-CCA and DUS Schisto POC-CCA methods respectively. The mean intensity of infection among infected participants was 86.3 eggs per gram of stool (EPG) ranging from 12.0 EPG to 824.0 EPG. The sensitivity of DUS Schisto POC-CCA and direct urine Schisto POC-CCA was 94.44% (95% *CI*: 89.15–99.74%) and 97.22% (95% *CI*: 93.43–100.00%) respectively. The DUS Schisto POC-CCA method had slightly higher specificity (66.85%) than direct urine Schisto POC-CCA method (63.48%). The accuracy of the DUS Schisto POC-CCA was found to be slightly high (74.80%, 95% *CI*: 68.94–79.06%) compared to that of direct urine Schisto POC-CCA (73.20%, 95% *CI*: 67.25–78.59%). There was good agreement between two laboratory technologists who performed the DUS Schisto POC-CCA method on similar samples (*k* = 0.80, 95% *CI*: 0.59–0.95).

**Conclusions:**

The DUS Schisto POC-CCA method had comparable *S. mansoni* detection accuracy to direct urine Schisto POC-CCA. This suggests that the method could be a potential alternative to direct urine Schisto POC-CCA for screening *S. mansoni* in resource-limited situations.

**Graphical Abstract:**

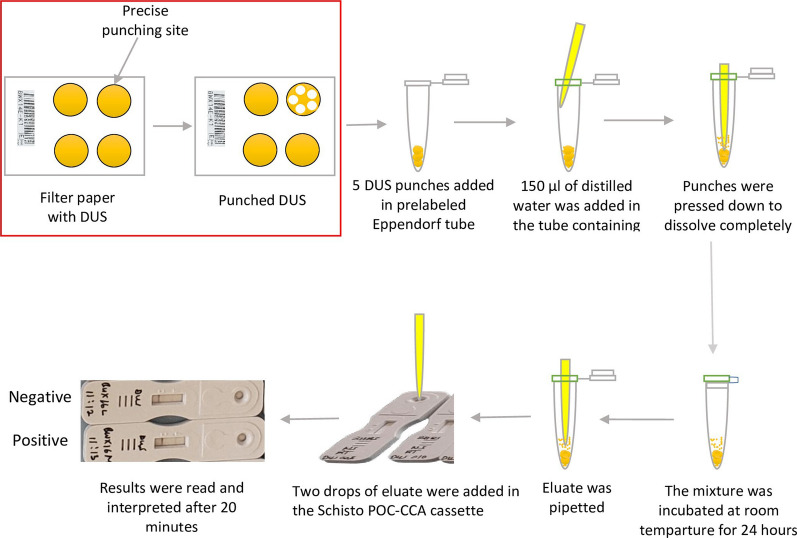

## Background

Diagnosis is one of critical components for surveillance and mapping of neglected tropical diseases (NTDs) [1]. The progressive reduction in the intensity and prevalence of NTDs may reduce the sensitivity and specificity of current diagnostic methods, causing them to fail to provide the point-of-care service required by control programs [2]. The majority of available methods necessitate the detection of parasite stages. However, as countries approach to the stage of NTDs elimination, infection rates fall, making these methods less sensitive in assessing transmission interruption [1]. This is the case for schistosomiasis, where the traditionally used diagnostic techniques, Kato-Katz thick smear (KK) for intestinal schistosomiasis and urine filtration for urogenital schistosomiasis have shown these pitfalls, especially in areas with low endemicity [3, 4]. Therefore, a highly sensitive and rapid diagnostic test is critical for effective schistosomiasis surveillance and mapping during the elimination stage [5].

More sensitive *Schistosoma* antigen detection diagnostic methods have recently been developed [3]. The most common methods rely on circulating antigens produced by adult worms. These antigens include circulating cathodic antigens (CCA) and circulating anodic antigens (CAA) [3, 6]. Because the CCA and CAA detection methods are more sensitive than microscopy, they provide more accurate information about *Schistosoma* infection prevalence [7, 8]. The World Health Organization currently recommends that control programs use point-of-care CCA cassettes (Schisto POC-CCA) to map the prevalence of *Schistosoma mansoni* [9]. Despite its high sensitivity and specificity, the CAA method cannot be used in the field because it requires laboratory equipment [7, 10]. Schisto POC-CCA cassettes, on the other hand, are sold commercially by few companies [11]. The presence of few manufacturers impedes the timely availability of the test cassettes [10]. Furthermore, the majority of schistosomiasis control programs rely on the support of their implementing partners (donors), and the implementation of their activities is heavily dependent on the availability of that support [12]. Furthermore, it may come a time as with the COVID-19 pandemic where industrial production become down and material transportation within and across continents become difficult. Therefore, the program may necessitate the use of low-cost sample collection and storage methods that could be used even in a resource-constrained setting with a high schistosomiasis burden, especially when time for data (specimen) collection is an integral part of program activities. This demand is driving the development of a simple and low-cost sampling technique for use during sample collection, transportation, storage and analysis in resource-constrained environments.

*Schistosoma* circulating antigens are stable and detectable in urine, blood, and serum samples [13, 14]. Urine samples have a number of advantages over blood and serum samples. Sample of urine can be kept for several days without the use of a cold chain. Urine collection is also noninvasive and does not require the use of trained medical personnel. Furthermore, a large volume can be collected easily [11]. With liquid urine, however, there is an increased risk of infection (leakage). Also, liquid urine is difficult to transport (due to the heavy load of urine containers) and store (due to the large amount of space required). A dried urine spot (DUS) using filter papers is considered simple and cost-effective when compared to liquid urine [15, 16]*.* Study was conducted to test various filter paper-based dried matrix method conditions for DUS collection and processing. The study revealed the recommended conditions that performed better for the collection of urine samples on filter paper and recovery of *S. mansoni* CCA from urine spotted on the filter papers [17]. The current study explore the accuracy and precision of the filter paper-based DUS method for the collection and detection of *S. mansoni* CCA using the recommended conditions described in a previous study.

## Methods

### Study design

A cross-sectional study was conducted between October and November 2022 using a paired comparative diagnostic test accuracy method to assess the accuracy and precision of the index method (DUS Schisto POC-CCA) and its comparator method (direct urine Schisto POC-CCA) for the collection of urine samples and detection of *S. mansoni* CCA.

### Sample collection site and population

**Fig. 1:**
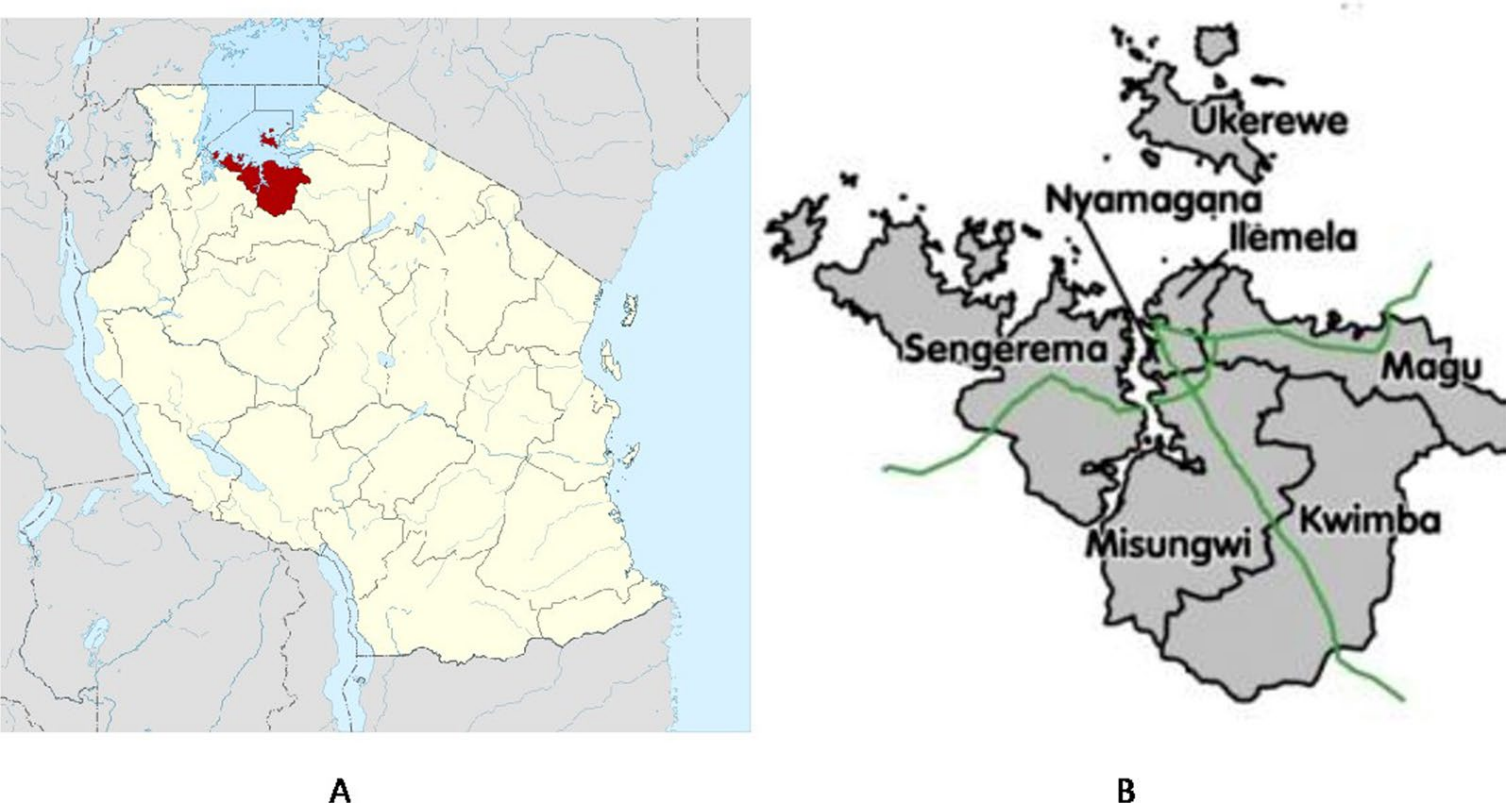
Map of Tanzania showing the Mwanza region (area highlighted red) (**A**) and that of the Mwanza region showing the location of the Sengerema district (**B**)

Samples for this study were collected from 250 primary school aged children (PSAC) in Sengerema district in the Mwanza region. The region is in northwestern Tanzania (Fig. 1), south of Lake Victoria, between latitudes 1° 30' and 3° 2' south of the equator and longitudes 31° 45' and 34° 10' east of the Greenwich meridian. Schistosomiasis is highly endemic in the region, particularly in communities along the shores of Lake Victoria. The prevalence of *S. mansoni* among PSAC in the region ranges from 2.9% to 94.5% [18]. Two primary schools with a previously reported high prevalence of *S. mansoni* were purposefully selected.

### Sample size estimation

The sample size for the paired comparative diagnostic test accuracy study was calculated based on Eqs. 1 and 2 as provided by Alonzo et al*.* [19].

1 $$nd={\left(\frac{{Z}^{\left(1-\beta \right)}+{Z}^{(1-\alpha *)}}{{\text{log}}(\frac{y}{S})}\right)}^{2}\left(\frac{(y+1)TPRe-2TPPR}{y(TPRe{)}^{2}}\right)$$

where *Z* is the score for a specific level of significance, 1– *β* is the study's power, α* is the significance level such that 1 − √(1- *α*), y is the relative true positive rate of a new test over the existing one, *s* is less than or equal to *y*, TPRe is the true positive rate of the existing test, TPPR is the proportion of diseased subjects tested positive by both tests (TPPR ≤ TPRe), and nd is the number of positive cases needed for this study. Assuming the study's power of 0.9, *α* is 0.05, *y* is 1.1, *s* is 0.9, TPRe (sensitivity) of the direct liquid urine method is 0.947 when duplicate KK method is the reference standard method [20], TPPR is the same as TPRe, which is 0.947, and nd is 164 PSAC.

Since

2 $$n = nd/p$$

where *p* denotes the expected prevalence and *n* denotes the needed sample size. Assuming an expected prevalence of 0.661 [20], the sample size *n* was 249.

### Sampling technique

The accuracy study used a multistage sampling technique to obtain a representative sample. Sampling was performed in a 3-stage cluster sampling technique where the 1st stage involved simple random selection of one endemic ward from the list of all wards in Sengerema district. The 2nd stage was the selection of primary schools from the list of all primary schools in a selected ward. Two schools were randomly selected in which 250 pupils were sampled. The total number of pupils in standards 3 and 4 in each school was used to determine the number of recruited pupils in each school and each class. Random sampling was used to select individual pupils to be included using pieces of paper either written recruit or left blank.

### Eligibility criteria

The study included PSAC who were in standard 3 or 4, attended school during the study period, permanent residents of the selected wards for at least 2 years, provided stool and urine samples, provided assent and head teachers signed informed consent. PSAC reported to be sick (apart from *S. mansoni*), who failed to provide stool and/or urine samples and who were not residents of the selected wards were excluded from the study.

### Data collection

#### Individual questionnaires

During the accuracy study, short interviews were conducted to collect sociodemographic information from primary PSAC. A simple questionnaire was used to collect information such as sex, age, class, and village of residency. An interview was conducted among PSAC who provided stool and urine samples only.

#### Parasitological procedures

Stool, liquid urine and saturated filter paper urine samples were collected from selected PSAC. Children were given a prelabeled [with identification number (ID)] wide-mouthed stool and urine containers for the collection of fresh stools and urine samples. They were asked to submit approximately 20 g and 20 ml of fresh stool and urine samples, respectively. From each received urine sample, the laboratory technologist saturated five prelabeled 2 × 3 inch filter (Whatman protein saver) papers with the respective liquid urine sample [17]. Then, the urine samples were further tested for *S. mansoni* CCA using direct liquid *Schistosoma* CCA testing using *Schistosoma* CCA cassette tests (Rapid Medical Diagnostics, Cape Town, South Africa; batch number 220701075), as per the manufacturer’s procedure manual. Trace result was classified as positive [21]. Due to the nature of urine (colorless to semi colorless) samples, filter papers with printed circles were used to easily identify the spots both immediately after application and after drying. Urine samples (both liquid and on filter paper) were entered into a sample registration book after they were received/prepared. The sample ID, volume and color were recorded. Saturated filter papers were transported to the National Institute for Medical Research (NIMR)—Mwanza Center parasitology laboratory and stored in a fly proof dry box overnight and processed as described below. The remaining liquid urine samples were added to Eppendorf tubes and transported and stored at Muhimbili University of Health and Allied Sciences (MUHAS)—Bagamoyo parasitology laboratory for future use.

Detection of *S. mansoni* CCA from a filter containing dried urine spots was performed at the NIMR—Mwanza Center parasitology laboratory using a procedure described in a previous publication [17]. Briefly, the filter papers containing DUS were moved from the fly proof dry box to the punching bench. After that, the precise location of the DUS on each filter paper was determined. Using a hand punching machine, 5 DUS disks (punches with 6 mm diameter each) were punched from each filter paper and placed in a separate prelabeled Eppendorf tube. The Eppendorf tube with the five punches was then filled with 150 µl of distilled water. The pointed end of an unused micropipette tip was pressed to the bottom of the tube to ensure that the five DUS punches were completely dissolved in distilled water. The tube was then incubated at room temperature for 24 h. With minor modifications, the detection of *S. mansoni* CCA was carried out in accordance with the Schisto POC-CCA procedure manual [21]. To summarize, all assay materials were brought to the working bench. The dissolved punches were pressed on one side of the Eppendorf tube with the pipette provided in the Schisto POC-CCA kits to separate the eluate from the punches and allow the pipette to reach the bottom of the tube. The pipette was then removed from the tube, squeezed, and reinserted into the tube with the tip touching the bottom. The eluate was allowed to fill by gently releasing the pipette. By gently squeezing the pipette, two drops of eluate (equivalent to 90–100 µl) were transferred into the circular well of the prelabeled Schisto POC-CCA test cassette. The eluate was allowed to completely absorb into the circular well's specimen pad. On the test cassette, the time for reading the results was then marked. The result was read and recorded in the results form as negative or positive twenty minutes after the sample was added to the circular well. Trace result was classified as positive [21].

Stool samples were recorded in a sample registration book. The sample ID, color, presence of blood or mucus, and consistency were all recorded. Stool samples were used to prepare duplicate KK thick smears after registration. The KK procedure described in the WHO bench aids for the diagnosis of intestinal parasites was used for the preparation of duplicate KK thick smear slides [22]. Duplicate slides were prepared for each stool sample. The smears were stored in a slide rack and transported to the NIMR-Mwanza parasitology laboratory for microscopic examination. The slides were examined by a well-experienced microscopist. Ten percent of the slides were examined by another experienced microscopist for quality control. The KK results was reported inform of number of *S. mansoni* eggs per gram of stool (EPG). The intensity of *S. mansoni* infection was classified based on the WHO guideline [22] as light intensity (1–99 EPG), moderate (100–400 EPG) and heavy (> 400 EPG).

#### Determination of reproducibility of the DUS method

A total of 40 DUS samples from PSAC were used for reproducibility testing. The samples were tested by two laboratory technologists in two different laboratories (MUHAS for laboratory technologist 1 and NIMR parasitology laboratories for laboratory technologist 2) to determine interrater reliability. The two laboratory technologists were asked to perform the test on the same day using the same described DUS procedure. The two laboratory technologists were blinded to the results of their colleague's test. Technologist 1 in the MUHAS parasitology laboratory was asked to repeat the procedure on the same samples three days after the first testing to determine intratester reliability.

### Data analysis

The collected data were cleaned prior to coding, entered, and then analyzed. The IBM SPSS Statistics for Windows (Version 23.0. Armonk, NY: IBM Corp, USA) was used for analysis of descriptive statistics such as frequencies, proportions and means. The same software was used to make cross tabulation and perform chi-square testing. SAS statistical software was used to calculate measures of accuracy, such as sensitivities, specificities and predictive values, and precision of the index test.

The sensitivities, specificities, negative predictive values and positive predictive values of the index test (DUS method) and comparator method (direct urine method) were computed using duplicate KK method as the reference standard method. The difference in measures of accuracy (sensitivity and specificity) between the DUS method and direct urine method were computed using the McNemar test. A *P* value < 0.05 was considered to indicate a statistically significant difference. Also, the sensitivities, specificities, negative predictive values and positive predictive values of the index method were calculated using the comparator method as the reference standard method.

The interrater agreement was calculated by comparing the results of two laboratory technologists who performed the test on the same samples. Intrarater agreement was calculated by comparing the first and second results obtained by a single laboratory technologist after repeating the procedure on the same samples. The strength of agreement for inter- and intrarater precisions were determined using Cohen’s kappa (*k*). The strength of agreement was classified as poor if *k* was ≤ 0.20, fair if *k* ranged between 0.21 and 0.40, moderate if *k* ranged between 0.41 and 0.60, good if *k* ranged between 0.61 and 0.80, and very good if *k* ranged between 0.81 and 1.00.

## Results

### Demographic characteristics of the study population

This study included 250 PSAC from two primary schools. There were 128 children from Kasomeko primary school (51.2%, 95% *CI*: 45.2–57.6%) and 122 from Nyamazugo primary school (48.8%, 95% *CI*: 42.4–54.8%). Males made up 54% (95% *CI*: 47.6–60.0%) of all participants. A total of 133 (53.2%, 95% *CI*: 47.2–58.8%) were in standard 3, and 117 (46.8%, 95% *CI*: 41.2–52.8%) were in standard 4. The mean age was 10.64 years (ranging from 8 to 15 years).

### Prevalence and intensity of *Schistosoma mansoni* infection among school children

**Fig. 2:**
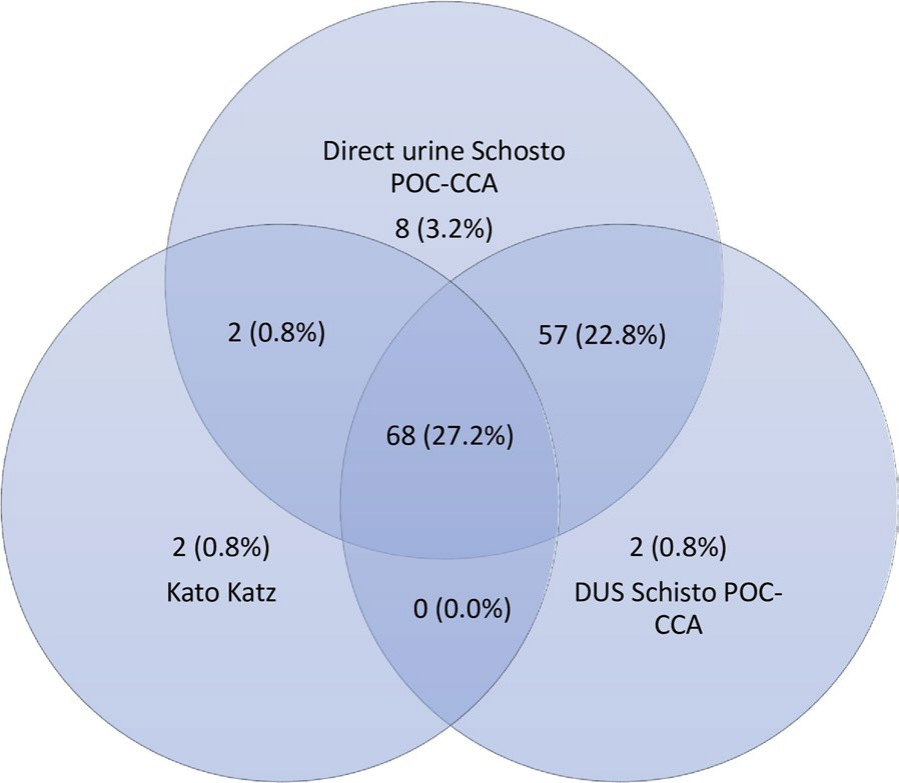
Venn diagram showing the number of *S. mansoni* infected school children as determined by each of the three diagnostic methods

Out of 250 PSAC, 139 (55.6%, 95% *CI*: 40.8–71.6%) tested positive with at least one of the three diagnostic methods. The *S. mansoni* prevalence by the KK method was 28.8% (95% *CI*: 23.6–34.8%), by the direct urine Schisto POC-CCA cassette method was 54.0% (95% *CI*: 48.0–60.4%), and by the DUS Schisto POC-CCA cassette method was 50.8% (95%* CI*: 44.8–57.6%). Sixty-eight PSAC tested positive for all three diagnostic methods; 57 tested positive for both the direct urine and DUS Schisto POC-CCA cassette methods, and two tested positive for both the KK and direct urine Schisto POC-CCA cassette methods (Fig. 2). Out of 72 *S. mansoni* infected PSAC as diagnosed by KK technique, 57 (79.2%) had light intensity of infection, 12 (16.7%) had moderate intensity of infection and three (4.2%) had heavy intensity of infection. The mean intensity of infection among infected participants was 86.3 EPG ranging from 12.0 EPG to 824.0 EPG.

### Accuracy of the dried urine spot and direct urine methods for the detection of *Schistosoma* circulating cathodic antigens

**Fig. 3:**
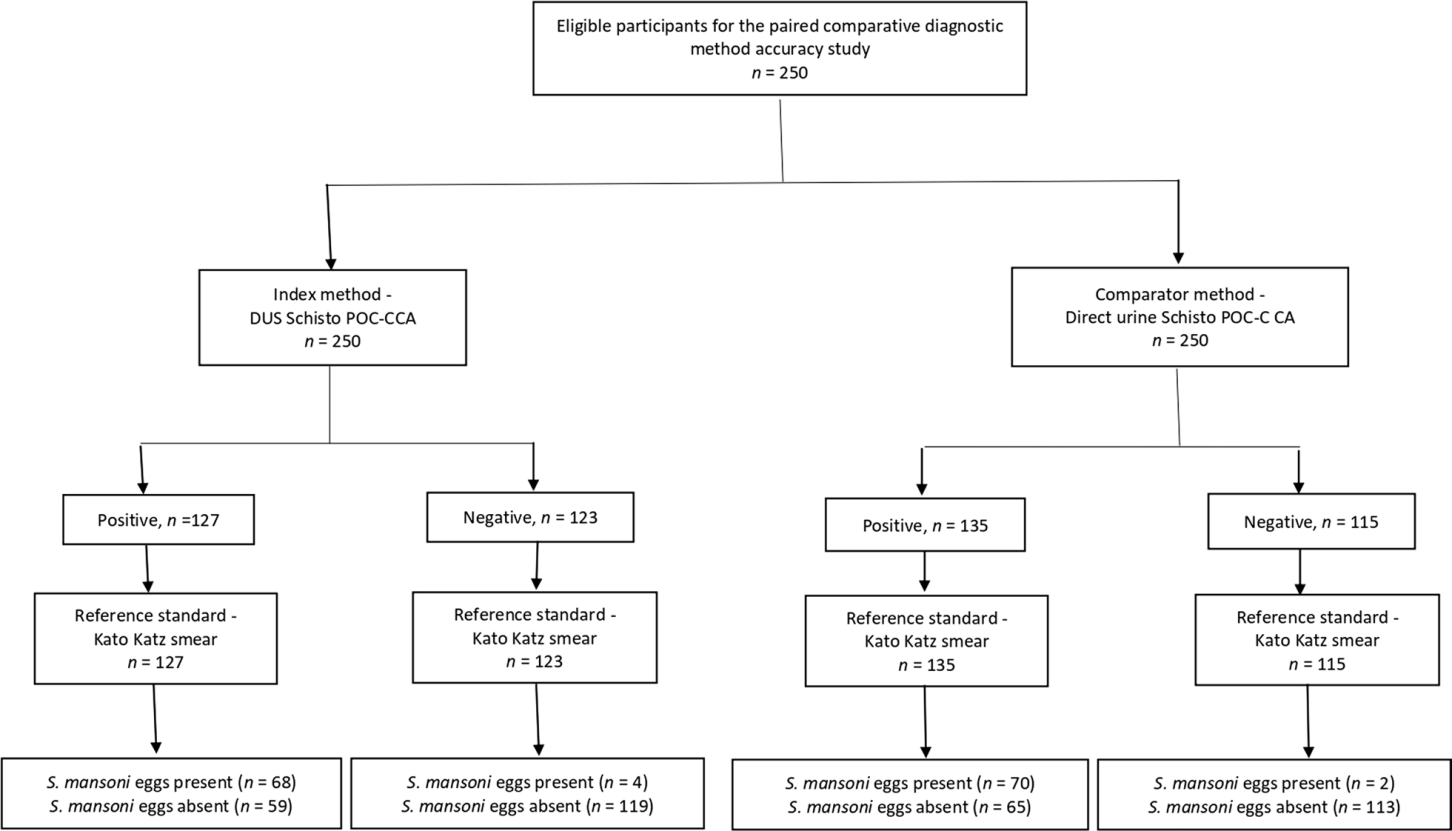
Diagrammatic description of the test results of the three *S. mansoni* diagnostic methods

Considering the duplicate KK method as the reference standard method, the true positive results were recorded in 70 participants for the direct urine Schisto POC-CCA method and 68 participants for the DUS Schisto POC-CCA method. The number of false positive cases was higher (65 cases) in the direct urine Schisto POC-CCA method than in the DUS Schisto POC-CCA method (59 cases). There were 119 true-negative cases for the DUS Schisto POC-CCA method and 113 for the direct urine Schisto POC-CCA method. Two false negative results were found for the direct urine Schisto POC-CCA method, while 2 false negative results were recorded for the DUS Schisto POC-CCA method (Fig. 3).

Table 1 presents values of various measures of accuracy recorded for both the direct urine Schisto POC-CCA method and the DUS Schisto POC-CCA method. The sensitivities of DUS Schisto POC-CCA and direct urine Schisto POC-CCA were 94.44% (95% *CI*: 89.15–99.74%) and 97.22% (95% *CI*: 93.43–100.00%), respectively. The DUS Schisto POC-CCA method had slightly higher specificity (66.85%) than the direct urine Schisto POC-CCA method (63.48%). An exact McNemar’s test determined that the difference in sensitivity and specificity between the two diagnostic test methods was not statistically significant (*P* = 0.500 for sensitivity and *P* = 0.109 for specificity). The DUS Schisto POC-CCA method had a slightly higher positive predictive value (53.54% vs. 51.85%) but a lower negative predictive value (96.75% vs. 98.26%) than the direct urine Schisto POC-CCA method. Of all tests performed by using the DUS Schisto POC-CCA method, 74.80% (95%* CI*: 68.94–79.06%) were accurate, while of those performed by using the direct urine Schisto POC-CCA, 73.20% (95%* CI*: 67.25–78.59%) were accurate.

**Table 1:** Measures of accuracy for DUS Schisto POC-CCA and direct urine Schisto POC-CCA methods

Measure of test accuracy	Direct urine POC-CCA		DUS POC-CCA	
	Value	95% *CI*	Value	95% *CI*
Sensitivity (%)	97.22	93.43–100.00	94.44	89.15–99.74
Specificity (%)	63.48	56.41–70.56	66.85	59.94–73.77
Negative predictive value (%)	98.26	93.48–99.55	96.75	91.94–98.73
Positive predictive value (%)	51.85	43.42–60.28	53.54	48.87–62.22
Accuracy (%)	73.20	67.25–78.59	74.80	68.94–79.06

The measures of accuracy were computed using duplicate Kato-Katz method as reference standard

*CI* confidence interval

When the measures of diagnostic accuracy of DUS Schisto POC-CCA were computed using direct urine Schisto POC-CCA as the reference standard method, there were 125 true-positive cases, 113 true-negative cases, 10 false-positive cases and 2 false-negative cases. The sensitivity and specificity of DUS Schisto POC-CCA were 92.59% (95% *CI*: 88.17–97.01%) and 98.26% (95%* CI*: 95.87–100.00%), respectively. The positive predictive value (PPV) was 98.43%, and the negative predictive value (NPV) was 91.87%. The test produced 95.20% of all test results accurately (Table 2).

**Table 2:** Measures of accuracy for DUS Schisto POC-CCA method

Measure of test accuracy	Value	95% *CI*
Sensitivity (%)	92.59	88.17–97.01
Specificity (%)	98.26	95.87–100.00
Negative predictive value (%)	91.87	87.04–96.70
Positive predictive value (%)	98.43	96.26–100.00
Accuracy (%)	95.20	91.77–97.50

The measures of accuracy were computed using direct urine Schisto POC-CCA method as reference standard

*CI* confidence interval

### Precision of the dried urine spot method for the detection of *Schistosoma* circulating cathodic antigens

Both laboratory technologists reported 19 samples as positive and 17 samples as negative (Table 3). The proportions of positive samples reported by laboratory technologists 1 and 2 were 55.0% (95% *CI*: 40.0–70.0%) and 50.0% (95% *CI*: 35.0–67.5%), respectively. The Cohen-Kappa test determined the level of agreement between the two laboratory technologists as good, with *k* = 0.80 (95% *CI*: 0.59–0.95).

**Table 3:** Cross tabulation of DUS Schisto POC-CCA test results reported by the two laboratory technologists

		Laboratory technologist 2		Total
		Positive	Negative	
Laboratory technologist 1	Positive	19	3	22
	Negative	1	17	18
Total		20	20	40

Laboratory technologist 2 reported 20 and 19 positive samples on the first and second testing occasions, respectively. The sample that tested differently was reported to be positive during the first testing and negative during the repeated testing. The proportion of positive samples during the first testing was 50.0% (95% *CI*: 35.0–67.5%), and after repeating the procedure, it was 47.5% (95% *CI*: 32.5–62.5%). The agreement between the first and repeated procedures was very good, with *k* = 0.95 (95% *CI*: 0.85–1.00).

## Discussion

The prevailing agreement indicates that POC-CCA demonstrates greater sensitivity in detecting *S. mansoni* infection when compared to the traditional KK method [23]. Several extensive studies endorse POC-CCA as a quick and uncomplicated test with heightened sensitivity [24]. Although POC-CCA cassettes exhibit remarkable sensitivity and specificity, they are often scarce in resource-constrained regions such as Tanzania. Consequently, there is an imperative need to create a straightforward and cost-effective alternative method that can be readily prepared in field conditions, demands minimal expertise, facilitates easy transport, and minimizes the risk of infection, unlike the current POC-CCA procedure requirement. To our knowledge, this is the first study to explore the accuracy and precision of the filter paper-based DUS method for the collection and detection of *S. mansoni* CCA.

Several studies have found that when the *S. mansoni* prevalence by KK method is greater than 50%, the POC-CCA and KK methods produce comparable results. When the prevalence by KK method is low, the POC-CCA prevalence can be many times higher. In areas where the POC-CCA prevalence is less than 30%, the KK prevalence appears to be less than 10%, if not zero [25]. These findings corroborate our findings where the prevalence of POC-CCA and DUS POC-CCA was 0.9- and 0.8-fold higher, respectively, than that of KK method. This may be due to the fact that, in schistosomiasis, immature worms during the acute phase or in recently reinfected cases may produce worm antigens (e.g., CCA) before eggs are excreted in the stool. This process may result in a positive result using the POC-CCA method and a negative result using the KK method [26]. Additionally, the low sensitivity of KK method can be attributed to the relatively small stool sample examined, the variability of *S. mansoni* eggs in a stool sample between days and the heterogeneous distribution of eggs within the stool sample [27–29].

Regarding the prevalence of *S. mansoni*, our findings are similar to those of Burundi [29], which showed a significantly higher prevalence of *S. mansoni* infection using the direct urine POC-CCA method as opposed to conventional microscopy of duplicate KK method. Additionally, the study that has been conducted in Côte d’Ivoire revealed a fivefold higher prevalence of *S. mansoni* (33.0%), whereas POC-CCA method with trace results considered negative resulted in a twofold higher prevalence (12.5%) compared with stool microscopy [30].

Our results showed that 57 (22.8%) PSAC were positive by both POC-CCA and DUS-CCA methods but negative by the KK method, and only 2 (0.8%) PSAC were positive by only KK method. The argument is that the POC-CCA and DUS POC-CCA methods are much more sensitive for diagnosing *S. mansoni* infection than the KK method. The possible reasons for positive POC-CCA and DUS POC-CCA methods and negative KK method results include infection with single-sex worms, infection with infertile worms, host anti-fecundity immunity and cross-reactivity of POC-CCA [31].

Our results also showed that when duplicate KK method is used as the reference standard method, the PPVs of both direct urine POC-CCA method and DUS POC-CCA method are low (51.85% and 53.54%, respectively). This could be due to the low sensitivity of the KK method, which has been consistently shown by different studies [31, 32], whereby the sensitivity of the POC-CCA method is compromised when it is applied in areas with low parasite loads, where the KK method is not able to capture all eggs for reasons described earlier. The higher sensitivity of the POC-CCA method may also be explained by taking into account findings from animal models. It has been showed that CCA levels for *S. mansoni* are detectable as early as 3 weeks after experimental infection, highlighting the potential to also capture infection status with juvenile stages not yet producing eggs [30]. However, it should be noted that false positive results for the direct urine POC-CCA method have been reported under some conditions such as in person with urinary tract infections, hematuria and pregnancy [33, 34]. This has been associated with the presence of a CCA polysaccharide structure that contains repeating units of Lewis-X trisaccharide molecules similar to that found in human cells [34, 35].

Nevertheless, the high NPVs for both POC-CCA methods indicates they have high accuracy in correctly identifying individuals who do not have the condition, and have low rate of false negatives. However, the high NPVs could further be validated by employing more sensitive diagnostic techniques (such as molecular methods for detection of parasite DNA) as reference standard method. Almost the same results were produced when direct urine POC-CCA method was used as a reference standard, the NPV of DUS POC-CCA method was 91.87%, and the specificity was 98.26%, which indicates that even when the direct urine POC-CCA method was used as a reference standard method, DUS POC-CCA method still has the ability to correctly identify individuals who do not have the condition and lower chances of producing false negatives. However, when the direct urine POC-CCA method was used as a reference standard method, PPV and sensitivity became very high compared with when the duplicate KK method was used as a reference standard method. This means that the two tests are highly correlated in identifying *S. mansoni* infections.

Despite the known low sensitivity of the reference standard (duplicate KK) method, both the DUS POC-CCA method and the direct urine POC-CCA method demonstrated relatively the same level of accuracy, indicating the potential of the DUS POC-CCA method as an alternative to the direct urine POC-CCA method. However, the low sensitivity of the duplicate KK method, on the other hand, should be considered as a limitation in computing the actual measures of accuracy for the two POC-CCA methods.

The Cohen-Kappa values indicate better agreement between the raters. In this context, it indicates the agreement between the technologists in classifying samples as positive or negative despite being conducted in two different settings. This indicates the good repeatability of the tests; however, more studies should be conducted to validate this.

The KK method which was used as reference standard has low sensitivity. The low sensitivity of the KK method may hinder the cross-validation with the index method (the DUS Schisto POC-CCA method). Due to the possibility of lack of robust cross-validation with a reference standard method, the true accuracy and precision of the DUS Schisto POC-CCA method might be challenging to ascertain conclusively.

## Conclusions

The current study assessed the reliability of using the DUS method for the detection of *S. mansoni* CCA in an endemic area in Sengerema District, northwestern Tanzania. The findings indicated that the DUS POC-CCA method performed almost similarly to the currently used direct urine POC-CCA method. The overall findings indicate that the DUS method provides promising results for becoming an alternative option for the collection of urine samples and the detection of *S. mansoni* CCA using POC-CCA cassettes. The method is sensitive and affordable, and may play an important role in schistosomiasis control programs, especially in resource-limited schistosomiasis endemic countries.

## Data Availability

The datasets used and/or analysed during the current study are available from the corresponding author on reasonable request.

## Abbreviations

CAA: Circulating anodic antigens
CCA: Circulating cathodic antigens
CI: Confidence interval
DUS: Dried urine spot
KK: Kato-Katz
MUHAS: Muhimbili University of Health and Allied Sciences
NIMR: National Institute for Medical Research
NPV: Negative predictive value
NTD: Neglected tropical diseases
POC: Point of care
PPV: Positive predictive value
PSAC: Primary school aged children
WHO: World Health Organization
